# Male breast cancer: clinicopathological features and prevalence of germline pathogenic variants

**DOI:** 10.1007/s10549-026-08062-9

**Published:** 2026-11-04

**Authors:** P. V. B. D. Cerqueira, F. A. B. Campos, S. M. Caputo, E. Rouleau, H. K. Mantovani, L. R. da Silva, A. L. Cicilini, S. M. Sanches, F. Makdissi, A. A. B. A. da Costa, M. De Brot, C. Osório, V. Castro Vasconcelos, V. Cordeiro de Lima, J. C. Casali da rocha, D. Carraro, G. Torrezan, E. Santana dos Santos

**Affiliations:** 1 Department of Oncology, Instituto De Assistência Médica Ao Servidor Público Estadual, Pedro de Toledo, 1800, São Paulo, 04039-000 Brasil; 2 A.C. Camargo Cancer Center, Tamandaré 753, São Paulo, SP 01525-001 Brazil; 3 UNICAMP - Universidade Estadual de Campinas, São Paulo, Brasil; 4 Department of Genetics, Institut Curie, Paris Sciences Lettre University, Paris, France; 5 Department of Genetics, Gustave Roussy, Paris, France; 6 Present Address: Department of Oncology, Instituto de Assistência Médica ao Servidor Público Estadual (IAMSPE), São Paulo, Brazil

**Keywords:** Breast cancer, Male breast cancer, Germline variants, Hereditary breast cancer

## Abstract

**Purpose:**

Male breast cancer is rare, and data on its clinicopathological and germline characteristics remain scarce. Our objective was to describe the clinicopathological profile of men with breast cancer and to estimate the prevalence of pathogenic germline variants in cancer predisposition genes.

**Methods:**

This multicenter retrospective study included adult men diagnosed with breast cancer between January 2000 and June 2022 at two institutions. Clinical and pathological data were obtained from medical records. Germline analysis was performed in patients without prior testing using DNA from biobanks or saliva samples and a custom multigene panel.

**Results:**

A total of 106 patients were included. Median age at diagnosis was 58.5 years (range, 33–84), and 64% reported a family history of cancer. Invasive ductal carcinoma was the predominant histological subtype (88%). Most tumors were grade 2 or 3 (90%) and hormone receptor-positive (93%). Hormone receptor-negative/HER2-positive disease was rare (1%), and triple-negative breast cancer accounted for 4% of cases. At diagnosis, 65% had stage II or III disease. Germline testing was performed in 55 patients, of whom 12 (22%) harbored pathogenic or likely pathogenic variants. The most frequently affected gene was *BRCA2* (11%), followed by *CHEK2* (5.5%), *MUTYH* (3.6%), and *RAD51C* (1.8%).

**Conclusion:**

Male breast cancer in this cohort was predominantly luminal, high-grade, and frequently diagnosed at advanced stages. The high prevalence of germline pathogenic variants, including among patients without a strong family history, supports broader implementation of genetic counseling and multigene testing in this population.

**Supplementary Information:**

The online version contains supplementary material available at https://doi.org/10.1007/s10549-026-08062-9.

## Introduction

Male breast cancer (MBC) is a rare malignancy, accounting for less than 1% of all breast cancers and approximately 0.5% of cancers in men [1–3]. Despite its low absolute incidence, rates have gradually increased over recent decades, likely reflecting population aging and greater recognition of risk factors associated with hyperestrogenism, including obesity, Klinefelter syndrome, and exogenous hormone exposure [4–9]. 

The epidemiology of MBC varies across geographic regions and populations, suggesting the influence of environmental, biological, and sociodemographic factors. Higher incidence rates have been reported in certain regions of Africa, potentially linked to chronic liver disease and altered estrogen metabolism [10]. In addition, disparities in incidence and outcomes have been observed across racial groups, with Black men showing both higher incidence and poorer prognosis than White men. These findings underscore the need for population-specific characterization of MBC [11–14]. 

Genetic predisposition plays a central role in MBC carcinogenesis. Pathogenic germline variants, particularly in BRCA2, represent one of the strongest risk factors, and the increasing use of multigene panel testing has expanded the detection of clinically actionable variants beyond *BRCA1/2* [15–17]. Accordingly, current guidelines recommend genetic counseling and germline testing for all men diagnosed with breast cancer, regardless of family history [18].

However, data on MBC remain limited. Available studies are small, frequently restricted to *BRCA1/2*, and rarely incorporate comprehensive multigene testing in consecutive patient series [19–25]. As a result, the clinicopathological spectrum and germline landscape h MBC remain insufficiently characterized.

In this study, we aimed to describe the clinicopathological features of men with breast cancer and to estimate the prevalence of pathogenic or likely pathogenic variants using a multigene approach in a non caucasian multicenter cohort, considering the hypothesis of a significant hereditary predisposition in male breast cancer and the need for multigene testing, since genetic mutations are not limited to BRCA2.

## Methods

### Study design and participating centers

This was a multicenter retrospective observational cohort study conducted at A.C. Camargo Cancer Center (coordinating center) and the University of Campinas (UNICAMP), Brazil. The study protocol was approved by the institutional review boards of both institutions. Eligible patients were identified between January 2000 and June 2022.

### Study population

Men aged > 17 years with histologically confirmed invasive or in situ breast carcinoma were included. Cases without institutional pathology review or with incomplete clinical records were excluded. Given the rarity of male breast cancer, we opted to use convenience sampling.

### Clinical and pathological data collection

Clinical and pathological data were obtained from electronic and paper medical records and recorded into a structured database (REDCap). Collected variables included age at diagnosis, family history of cancer, history of second primary malignancies, histological subtype (World Health Organization classification), histological grade (Nottingham system), and clinical stage at diagnosis according to the American Joint Committee on Cancer (AJCC).

Tumor biomarkers included hormone receptor status, HER2 expression, and Ki−67. Additional variables comprised locoregional invasion, surgical management, systemic therapies, and radiotherapy. Follow-up data included the site of recurrence and vital status at last follow-up.

### Germline analysis

Patients with available constitutional DNA (biobank-derived or saliva samples) underwent germline testing using multigene panels for hereditary cancer predisposition (Supplementary Appendix 1). Target enrichment was performed according to institutional protocols, followed by next-generation sequencing on NextSeq platform (Illumina). The panels included high- and moderate-penetrance cancer susceptibility genes, as detailed in the supplementary material.

### Variant classification

Sequencing reads were aligned and processed using a validated pipeline at the A.C. Camargo Genomics Laboratory. Variants were filtered based on quality metrics, sequencing depth, and allele frequency consistent with germline origin. Variant annotation incorporated the international and population-specific databases, including ExAC, ESP6500, ABraOM, ClinVar, LOVD, IARC. Variants were classified according to ACMG/AMP guidelines [26]. Variants of uncertain significance (VUS) were excluded from the primary analyses.

### Statistical analysis

Continuous variables were summarized as mean (standard deviation) or median (interquartile range), as appropriate. Categorical variables were reported as frequencies and proportions. Comparisons between groups were performed using Fisher’s exact test or Student’s t test, as appropriate.

Overall survival (OS) was defined as the time from diagnosis to death from any cause or last follow-up and estimated using the Kaplan–Meier method, and comparisons were performed using the log-rank test. Prognostic factors were evaluated using univariate and multivariate Cox regression models. The proportional hazards assumption was assessed using Schoenfeld residuals. All statistical tests were two-sided, with a significance level of *p* < 0.05. Analyses were performed using SPSS version 25 (IBM Corp.).

## Results

### Clinical and pathological findings

The cohort comprised 106 patients, of whom 71 (67%) were treated at A.C. Camargo Cancer Center and 35 (33%) at UNICAMP. In 37 cases (35%), the diagnosis occurred before 2010. The median age at diagnosis was 58.5 years (range, 33–84). A family history of cancer was reported in 64% of patients, including breast cancer in 32%, while no cases of male breast cancer were reported. Thirteen patients (12.3%) had a second primary malignancy, most commonly prostate cancer (12 of 13 cases).

Invasive ductal carcinoma was the predominant histological subtype (88%). Ductal carcinoma in situ (DCIS) and invasive lobular carcinoma were rare (1.9% and 1.0%, respectively). Most tumors were histological grade 2 or 3 (88.7%) and hormone receptor-positive (93%), consistent with a predominantly luminal phenotype. HER2-positive/hormone receptor-negative disease was uncommon (0.9%), and triple-negative breast cancer accounted for 3.9% of cases. At diagnosis, 89% of patients had localized or locally advanced disease, while 9.4% presented with metastatic disease, predominantly involving bone (8.5%) (Table 1).

**Table 1:** Clinical and anatomopathological characteristics of patients in this cohort

Variable	*n* = 106 (100%)
**Age (years)**,** median (variation)**	58.5 (33–84)
**Oncology family history**	
Yes	68 (64)
No	36 (34)
NA	2 (2)
**Breast cancer family history**	34 (32)
First grade	13 (12.3)
Second grade	17 (16)
Third grade	12 (11,3)
**Person history of other primary neoplasm**	
Yes	13 (12.3)
No	65 (61.3)
NA	28 (26.4)
**Histologic subtype**	
Invasive ductal carcinoma	93 (87.7)
Ductal carcinoma in situ	2 (1.9)
Invasive lobular carcinoma	1 (1)
Others	10 (9.4)
**Histologic grade**	
1	2 (2)
2	53 (50)
3	41 (38.7)
NA	10 (9.4)
**Immunohistochemical expression**	
Hormone receptor positive/HER2 negative	82 (77.3)
Hormone receptor positive/HER2 positive	17 (16)
Hormone receptor negative/HER2 positive	1 (0.9)
Hormone receptor negative/HER2 negative	4 (3.9)
NA	2 (1.9)
**Clinical staging**	
I	24 (23.5)
II	35 (33)
III	34 (32)
IV	10 (9.4)
NA	3 (3)
**Metastatic site**	
Lung	1 (0.9)
Bone	9 (8.5)
Non-regional lymph nodes	4 (3.8)
**Death number**	20 (18.8)

*12 patients with a history of prostate cancer (1 of whom also had a history of melanoma, 1 with bladder cancer, and 1 with colon cancer); 1 patient with a history of pancreatic cancer

NA = Not available

### Treatment patterns

All patients with non- metastatic disease, including those with DCIS, underwent surgical treatment. Total mastectomy was the most frequently performed procedure (85.8%), and axillary lymph node dissection was carried out in 63.2% of cases (Table S1).

Among patients with localized disease, 66% received chemotherapy, administered in the adjuvant 54% or neoadjuvant (12%) setting. The most used regimens were doxorubicin plus cyclophosphamide (18.9%) and doxorubicin plus cyclophosphamide followed by weekly paclitaxel (23.6%). Adjuvant radiotherapy was delivered to 48% of patients, and endocrine therapy with tamoxifen was used in 70% of cases.

### Germline findings

Germline testing was performed in 55 patients (52%) using DNA from biobanks (*n* = 15), saliva samples (*n* = 38), or prior clinical testing (*n* = 2). The remaining 51 patients (48%) were not tested due to the lack of available biological material. A table comparing the baseline characteristics of tested versus untested patients can be found in Table S2.

Pathogenic or likely pathogenic germline variants were identified in 12 patients (21.8%), including *BRCA2* (11%), *CHEK2* (5.5%), *MUTYH* (3.6%), and *RAD51C* (1.8%) (Table 2). The presence of the mutation did not result in differences in age (*p* = 0.6), family history (*p* = 0.9), histological grade (*p* = 0.9), staging (*p* = 0.3), molecular subtype (*p* = 0.8), or overall survival (*p* = 0.37). Variants of uncertain significance were detected in 25 patients (45.5%) (Supplementary Appendix 2). Copy number variations analysis was performed in 24 patients (43.6%), with no alterations identified.

**Table 2:** Detailed molecular findings from 12 patients carrying pathogenic/probably pathogenic germline variants

Patient	Panel size (genes)	Pathogenic/probably pathogenic variants			Variant of uncertain meaning
		Gene	Variant*	Protein	
1	27	CHEK2	c.349 A > G	p.Arg117Gly	Negative
2	94	RAD51C	c.93del	p.Phe32fs	NM_000057.4(BLM): c.599 C > A; p.(Thr200Lys) | NM_004364.4(CEBPA): c.1018G > A (p.Gly340Ser)
3	26	CHEK2	c.1427 C > T	p.Thr476Met	NM_004360.5(CDH1):c.949T > C (p.Phe317Leu)
4	26	BRCA2	c.2808_2811del	p.Ala938Profs	NM_000059.4(BRCA2):c.6988 A > G (p.Ile2330Val)
5	26	MUTYH	c.1063del	p.Ala357fs	Negative
6	26	BRCA2	c.7618−10T > G	-	Negative
7	26	BRCA2	c.2T > G	p.Met1Arg	Negative
8	26	BRCA2	c.2812_2815del	p.Ala938fs	NM_000059.3(BRCA2):c.3220 A > T (p.Ser1074Cys); NM_007194.4(CHEK2):c.542G > A (p.Arg181His)
9	27	BRCA2	c.5946del	p.Ser1982fs	Negative
10	26	MUTYH	c.452 A > G	p.Tyr151Cys	NM_000059.4(BRCA2):c.811G > A (p.Gly271Arg)
11	26	CHEK2	c.1036 C > T	p.Arg346Cys	Negative
12	144	BRCA2	c.8878 C > T	p.Gln2960Ter	Negative

*All variants found are in heterozygotes

Among carriers of pathogenic/likely pathogenic variants, the median age at diagnosis was 62.5 years (range, 38–69). Tumors were predominantly invasive ductal carcinoma, luminal subtype, and histological grade 2 or 3. No statistically significant association was observed between the presence of pathogenic/likely pathogenic variants and family history of breast cancer (21% vs. 5.7%; *p* = 0.16) or the occurrence of second primary malignancies (*p* = 0.74).

### Survival analysis

**Fig. 1:**
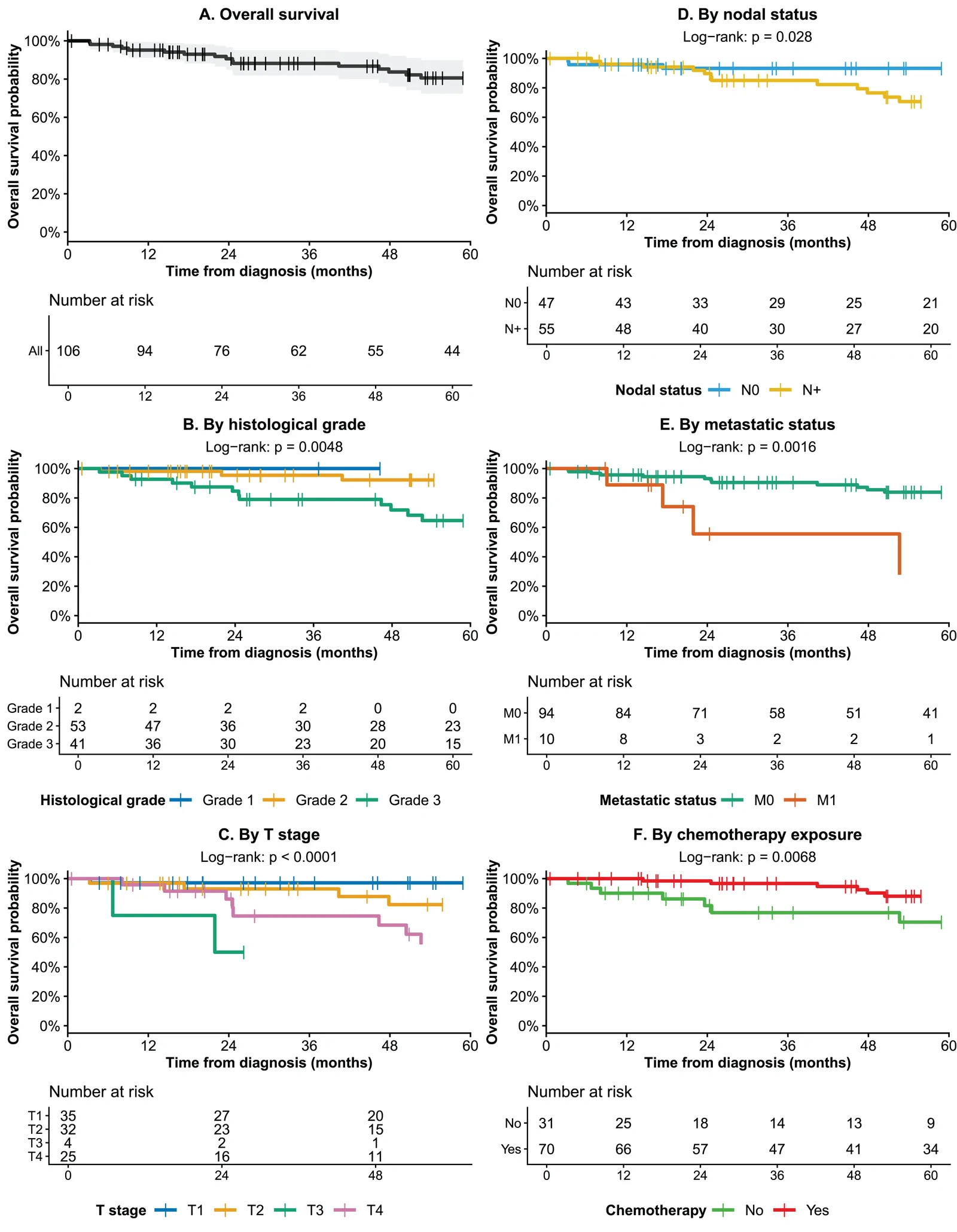
Overall survival Kaplan-Meier’s curves. Entire cohort (**A**) and stratified according to variables of interest: (**B**) Histological grade, (**C**) Stage T, (**D**) Stage N, (**E**) Stage M, (**F**) Neo/adjuvant chemotherapy

During the median follow-up of 64.5 months, 20 deaths were observed among the 106 patients (Table 1) the median overall survival was not reached (Fig. 1), and the estimated 5-year overall survival rate was 80.6%. In univariate analysis, poorer overall survival was associated with histological grade 3 (*p* = 0.005), larger tumor size (*p* < 0.001), lymph node involvement (*p* = 0.02), presence of metastases at diagnosis (*p* = 0.002), and having received neoadjuvant/adjuvant chemotherapy (*p* = 0.007) (Table S3).

In multivariate Cox regression analysis, histological grade 3 (HR 7.6; 95% CI, 1.6–34.7; *p* = 0.009), lymph node involvement (HR 6.4; 95% CI, 1.4–29.3; *p* = 0.017), and neoadjuvant/adjuvant chemotherapy (HR 8.7; 95% CI, 2.7–28.8; *p* = 0.0001) remained independently associated with mortality (Table S3).

## Discussion

This study assembled a consecutive cohort of 106 men with breast cancer treated at two institutions, describing their clinicopathological characteristics, treatment patterns, and the prevalence of germline variants in a subset of 55 patients. To our knowledge, this represents one of the largest series combining detailed clinicopathological characterization with multigene (beyond BRCA1/2) germline testing in an admixed, non-Caucasian, Latin American MBC population — a group markedly underrepresented in prior series, which have been predominantly European- or North American-derived. addressing an important gap in the literature (Table S5) [19–21, 27–32]. 

Family history of cancer was common, reinforcing the contribution of hereditary predisposition. However, family history alone proved to be an imperfect predictor of germline risk, was not significantly associated with the presence of pathogenic or likely pathogenic germline variants, in line with previous studies in male breast cancer [33–37]. 

From a pathological standpoint, our findings reflect the classical phenotype of male breast cancer: predominance of invasive ductal carcinoma, a high proportion of hormone receptor–positive tumors, and a low frequency of triple-negative disease. [33, 38, 39] More than 40% of patients presented with stage III or IV disease at diagnosis, underscoring the issue of delayed diagnosis in men. The absence of screening programs, low clinical suspicion, and the stigma associated with the disease likely contribute to this pattern.[10, 40–44].

Treatment approaches in this cohort were largely aligned with current practice, which is predominantly extrapolated from female breast cancer [4]. Most patients underwent total mastectomy, frequently combined with axillary dissection, and received adjuvant or neoadjuvant chemotherapy, radiotherapy, and endocrine therapy with tamoxifen (Table S1, S4) [45–53]. 

The 5-year overall survival rate was 80.6%, with median survival not reached after a median follow-up of 64.5 months. As expected, adverse prognostic factors included higher histological grade and lymph node involvement. The association between chemotherapy and worse outcomes likely reflects indication bias, as this treatment is preferentially administered to patients with more aggressive and advanced disease.Therefore, chemotherapy should be interpreted as a marker of disease aggressiveness or advanced stage, rather than as a prognostic factor. Survival outcomes were comparable to those reported in large population-based datasets, including SEER analyses [54]. A key strength of this study is the multigene germline evaluation. Among tested patients, 21.8% harbored pathogenic or likely pathogenic variants, predominantly in *BRCA2*, followed by *CHEK2*,* MUTYH*, and *RAD51C*. These findings are consistent with the reported range in the literature and reinforce that hereditary predisposition in male breast cancer extends beyond *BRCA2* (Table S5) [55]. 

The predominance of BRCA2 variants aligns with international data. Although the sample size limits robust gene-specific analyses, the observed tumor phenotype among *BRCA2*-carriers was broadly consistent with prior reports (Table S6). Interestingly, half of the patients with *BRCA2* variants showed HER2 overexpression, suggesting that tumor phenotype in men may be heterogeneous even within the same germline context (Table 3) [16, 27–32, 56–61]. 

**Table 3:** Clinical and pathological characteristics of patients carrying pathogenic/probably pathogenic variants

Patient	Altered gene	Personal history of a second cancer	Family history of cancer	Age at diagnosis	Tumor histology	Histologic grade	Molecular subtype	Clinical staging
1	CHEK2	No	Sister: Breast cancer, paternal aunt: Breast cancer, 2 paternal nieces: Breast cancer, paternal uncle: Head and neck cancer, paternal uncle: Bladder cancer, paternal aunt: Skin cancer	62	IDC	2	Luminal B	III
2	RAD51C	No	Brother: Pancreatic cancer, maternal aunt: Lung cancer, maternal cousin: Breast cancer, maternal uncle: Laryngeal cancer, maternal cousin: Breast cancer.	59	IDC	2	Luminal B HER2+	II
3	CHEK2	No	Maternal uncle: Lung cancer, paternal cousin: Leukemia	66	IDC	3	Luminal B	I
4	BRCA2	No	No	44	IDC	3	Luminal B	III
5	MUTYH	No	No	67	IDC	2	Luminal B	II
6	BRCA2	No	No	68	IDC	2	Luminal B HER2+	IV
7	BRCA2	Yes^a^	Father: Prostate cancer, brother: Prostate cancer, sister: Breast cancer	65	IDC	2	Luminal B	II
8	BRCA2	No	Father: Pancreatic cancer, sister: Breast cancer, cousin: Breast cancer, cousin: Stomach cancer	59	IDC	NI	Luminal B	III
9	BRCA2	No	Father: Pancreatic cancer, sister: Uterine cancer, maternal grandmother: Breast cancer	63	IDC	3	Luminal B	II
10	MUTYH	No	Paternal aunt: Breast cancer	52	IDC	3	Luminal B	III
11	CHEK2	No	Mother: breast cancer, sister: breast cancer	69	IDC	NI	Luminal B	I
12	BRCA2	No	Paternal grandmother: breast cancer, paternal aunt: breast cancer, maternal uncle: bladder cancer, father: cancer of unknown primary site, mother: colon cancer	38	IDC	2	Luminal B HER2+	II

IDC: Invasive ductal carcinoma

a: Patient with a personal history of prostate carcinoma and bowel adenocarcinoma

The detection of variants in CHEK2, MUTYH, and RAD51C further highlights the genetic heterogeneity of MBC and supports the clinical utility of multigene panel tasting. Although MUTYH is classically associated with autosomal recessive colorectal polyposis/cancer risk, monoallelic (heterozygous) pathogenic variants have been reported to confer a modest and maybe potentially clinically relevant increase in breast cancer risk, including in male breast cancer series using multigene panels. We now discuss the implications of this finding for genetic counseling (e.g., relevance for colorectal cancer surveillance in carriers and relatives) and note that larger, multigene-panel-basedtion may be underrecognized when testing is restricted to BRCA1/2. We now discuss the implications of this finding for genetic counseling (e.g., relevance for colorectal cancer surveillance in carriers and relatives) and note that larger, multigene-panel-based series are needed to clarify penetrance estimates specifically in men [57, 62–69]. 

For decades, VUS has become a real problem in medical genetics and precision medicine. Internationally, consortia have been formed to address this issue, particularly with regard to the *BRCA1* and *BRCA2* genes [70]. The high rate of VUS (45.5%) is a likely consequence of the underrepresentation of admixed Latin American/Brazilian populations in the reference population databases (e.g., ClinVar) used for variant classification. Furthermore, the practical implications of a high VUS burden for genetic counseling include the risk of over-interpretation by non-specialists and the need for periodic reclassification as population-specific data accumulate. The importance of incorporating Latin America-specific allele frequency databases into future variant classification workflows to reduce this disparity is also highlighted.

This study has several limitations, including the retrospective design, testing of only a portion of the cohort, and the number of mutated patients. The reason for not testing the entire cohort was the unavailability of biological material, rather than a systematic clinical criterion; this reduces, but does not eliminate clinicopathological selection bias. The small absolute number of carriers of pathogenic/probably pathogenic variants (*n* = 12) limits the statistical power to detect associations between mutation status and clinicopathological variables or survival outcomes; therefore, the reported absence of association should be interpreted with caution, and not as evidence of a true absence of effect. In the survival analysis, the histological grade 1 subgroup comprised very few patients, which limits the precision of the corresponding Kaplan-Meier estimate and may affect the reliability of comparisons involving this stratum; results for this subgroup should therefore be interpreted as hypothesis-generating rather than conclusive.

Taken together, these findings support the view of MBC as a biologically distinct entity with specific clinical and genetic implications. The combination of frequent hormone receptor-positive disease, delayed diagnosis, and a substantial prevalence of germline variants strongly supports referral for genetic counseling and multigene testing for all men diagnosed with breast cancer. Even a modest non-BRCA yield (4/55, 7.3%, of tested patients; 4/12, 33%, of all pathogenic/likely pathogenic carriers in this cohort) is clinically meaningful at the individual-patient level, given the actionability of CHEK2 and RAD51C findings for cascade testing in relatives.

From a therapeutic perspective, the identification of *BRCA2* and *RAD51C* variants has direct clinical implications, particularly regarding eligibility for PARP inhibitors in advanced disease. Although prospective data in men remain limited, these findings highlight the importance of germline testing not only for familial risk assessment but also for treatment personalization.

## Conclusion

In this cohort, male breast cancer was predominantly invasive ductal, hormone receptor-positive, and of intermediate to high histological grade. The disease was frequently diagnosed at advanced stages, reinforcing the ongoing challenge of delayed diagnosis in men. One-fifth of patients who underwent germline testing harbored pathogenic or likely pathogenic variants, predominantly in *BRCA2*, but also involving genes such as *CHEK2*, *MUTYH*, and *RAD51C*. These findings confirm that hereditary predisposition to male breast cancer is not limited to BRCA2, and support the use of BRCA-based genetic testing for all men, as well as multigene testing when available.

Our results support universal genetic testing for all men diagnosed with breast cancer. In resource-limited settings, prioritizing *BRCA2* testing may be a pragmatic approach; however, when feasible, multigene panels should be considered for their broader clinical relevance.

Further studies in larger and more diverse populations are needed to refine the role of specific genes and to optimize genetic testing strategies in male breast cancer.

## Supplementary Information

Below is the link to the electronic supplementary material.

Supplementary Material 1

## Data Availability

The datasets generated during and/or analyzed during the current study are not publicly available due to determination by the institutional ethics committee, but are available from the corresponding author on reasonable request.

## References

[1] Siegel RL, Miller KD, Wagle NS, Jemal A (2023) Cancer statistics, 2023. CA Cancer J Clin 73(1):17–48 10.3322/caac.21763 36633525

[2] Miao H, Verkooijen HM, Chia KS et al (2011) Incidence and outcome of male breast cancer: an international population-based study. J Clin Oncol 29(33):4381–4386 10.1200/JCO.2011.36.8902 21969512

[3] Hong JH, Ha KS, Jung YH et al (2016) Clinical features of male breast cancer: experiences from seven institutions over 20 years. Cancer Res Treat 48(4):1389–1398 10.4143/crt.2015.410 PMC5080810 27121722

[4] Hassett MJ, Somerfield MR, Baker ER et al (2020) Management of male breast cancer: ASCO guideline. J Clin Oncol 38(16):1849–1863 10.1200/JCO.19.03120 32058842

[5] White J, Kearins O, Dodwell D et al (2011) Male breast carcinoma: increased awareness needed. Breast Cancer Res 13(5):219 10.1186/bcr2930 PMC3262194 22017761

[6] Krapcho M et al (2016) SEER Cancer Statistics Review, 1975–2013. Bethesda (MD): National Cancer Institute; Available from: https://seer.cancer.gov/csr/1975_2013/

[7] Speirs V, Shaaban AM (2009) The rising incidence of male breast cancer. Breast Cancer Res Treat 115(2):429–430 10.1007/s10549-008-0053-y 18478326

[8] Sarmiento S, McColl M, Musavi L et al (2020) Male breast cancer: a closer look at patient and tumor characteristics and factors that affect survival using the National Cancer Database. Breast Cancer Res Treat 180(2):471–479 10.1007/s10549-020-05556-y 32040687

[9] Reddington R, Galer M, Hagedorn A et al (2020) Incidence of male breast cancer in Scotland over a twenty-five-year period (1992–2017). Eur J Surg Oncol 46(8):1546–1550 10.1016/j.ejso.2020.01.009 31955992

[10] Ottini L, Palli D, Rizzo S et al (2010) Male breast cancer. Crit Rev Oncol Hematol 73:141–155 10.1016/j.critrevonc.2009.04.003 19427229

[11] Anderson WF, Althuis MD, Brinton LA et al (2004) Is male breast cancer similar or different than female breast cancer? Breast Cancer Res Treat 83(1):77–86 10.1023/B:BREA.0000010701.08825.2d 14997057

[12] Howlader N, Noone AM, Krapcho M et al (2020) SEER Cancer Statistics Review, 1975–2017. Bethesda (MD): National Cancer Institute; Available from: https://seer.cancer.gov/csr/1975_2017/

[13] O’Malley C, Shema S, White E et al (2005) Incidence of male breast cancer in California, 1988–2000. Breast Cancer Res Treat 93:145–150 10.1007/s10549-005-4517-z 16187234

[14] Sineshaw HM, Freedman RA, Ward EM et al (2015) Black/white disparities in male breast cancer. J Clin Oncol 33(21):2337–2344 10.1200/JCO.2014.60.5584 25940726

[15] Giordano SH (2018) Breast cancer in men. N Engl J Med 378(24):2311–2320 10.1056/NEJMra1707939 29897847

[16] Tung N, Battelli C, Allen B et al (2015) Frequency of mutations in individuals with breast cancer referred for BRCA1 and BRCA2 testing using next-generation sequencing with a 25-gene panel. Cancer 121(1):25–33 10.1002/cncr.29010 25186627

[17] Campos FAB, Rouleau E, Torrezan GT et al (2021) Genetic landscape of male breast cancer. Cancers (Basel) 13(14):3535 10.3390/cancers13143535 PMC8305894 34298749

[18] Forbes C, Fayter D, de Kock S, Quek RGW (2019) A systematic review of international guidelines for BRCA-mutated breast cancer. Cancer Manag Res 11:2321–2337 10.2147/CMAR.S189627 PMC6434912 30962720

[19] Barbosa RTDA, Vertamatti MAF, Arab C et al (2018) Breast disease in men in São Paulo, Brazil. Breast J 24(2):216–218 10.1111/tbj.12879 28741872

[20] Thuler LCS, Aguiar SS, Rapozo D et al (2021) Male breast cancer in Brazil: a cohort study. Breast J 27(1):95–98 10.1111/tbj.14120 33258519

[21] Maselli-Schoueri JH, Affonso-Kaufman FA, De Melo Sette CV et al (2019) Mortality trends of male breast cancer in Brazil. BMC Cancer 19:23 10.1186/s12885-018-5261-1 PMC6323830 30616526

[22] Alemar B, Gregório C, Herzog J et al (2017) BRCA profile in Southern Brazil. PLoS ONE 12(11):e0187630 10.1371/journal.pone.0187630 PMC5697861 29161300

[23] Carraro DM, Koike Folgueira MAA, Lisboa BC et al (2013) BRCA1/2 and TP53 mutations in Brazil. PLoS ONE 8(3):e57581 10.1371/journal.pone.0057581 PMC3586086 23469205

[24] Silva FC, Lisboa BC, Figueiredo MC et al (2014) Hereditary breast cancer in Brazilian patients. BMC Med Genet 15:55 10.1186/1471-2350-15-55 PMC4038072 24884479

[25] Fernandes GC, Michelli RAD, Galvão HCR et al (2016) BRCA mutations in Brazilian population. Oncotarget 7(49):80465–80481 10.18632/oncotarget.12610 PMC5348334 27741520

[26] Parsons MT, de la Hoya M, Richardson ME et al (2024) ACMG/AMP variant classification recommendations for BRCA1/2. Am J Hum Genet 111(9):2044–2058 10.1016/j.ajhg.2024.07.013 PMC11393667 39142283

[27] Tedaldi G, Tebaldi M, Zampiga V et al (2020) Multigene panel testing in male breast cancer. Diagnostics (Basel) 10(5):269 10.3390/diagnostics10050269 PMC7277207 32365798

[28] Gaddam S, Heller SL, Babb JS, Gao Y (2020) Screening recommendations in high-risk men undergoing genetic testing. Clin Breast Cancer 20:1–6 10.1016/j.clbc.2020.07.014 32828665

[29] Scarpitta R, Zanna I, Aretini P et al (2019) Germline investigation in male breast cancer using next-generation sequencing. Breast Cancer Res Treat 178:557–564 10.1007/s10549-019-05429-z 31512090

[30] Fostira F, Saloustros E, Apostolou P et al (2018) Non-BRCA germline mutations in male breast cancer. Breast Cancer Res Treat 169(1):105–113 10.1007/s10549-018-4661-x 29335925

[31] Vogel Postula KJ, Andolina LM, Theobald K et al (2018) Multigene hereditary cancer panels in male breast cancer. Cancer Res 78(4 Suppl):PD7–11

[32] Susswein LR, Marshall ML, Nusbaum R et al (2016) Pathogenic variant prevalence in cancer panel testing. Genet Med 18(8):823–832 10.1038/gim.2015.166 PMC4985612 26681312

[33] Thuler LCS, Bergmann A (2015) Male breast cancer in Brazil. Aging Male 18(2):118–123 10.3109/13685538.2014.922532 24871582

[34] Rosenblatt KA, Thomas DB, McTiernan A et al (1991) Breast cancer in men: aspects of familial aggregation. J Natl Cancer Inst 83(12):849–854 10.1093/jnci/83.12.849 2061945

[35] Lesueur F, Mebirouk N, Jiao Y et al (2018) GEMO: a national resource to study genetic modifiers of breast and ovarian cancer risk. Front Oncol 8:490 10.3389/fonc.2018.00490 PMC6220051 30430080

[36] Brinton LA, Richesson DA, Gierach GL et al (2008) Prospective evaluation of risk factors for male breast cancer. J Natl Cancer Inst 100(20):1477–1481 10.1093/jnci/djn329 PMC2720728 18840816

[37] Calip GS, Kidd J, Bernhisel R et al (2020) Family history of breast cancer in men with non-BRCA disease. Breast Cancer Res Treat 10.1007/s10549-020-05922-w PMC7855353 32918117

[38] Cardoso F, Bartlett JMS, Slaets L et al (2018) Characterization of male breast cancer: results of the EORTC international program. Ann Oncol 29(2):405–417 10.1093/annonc/mdx651 PMC5834077 29092024

[39] Humphries MP, Rajan SS, Honarpisheh H et al (2017) Biomarker characterization in male breast cancer. Sci Rep 7:45293 10.1038/srep45293 PMC5368596 28350011

[40] Ishii T, Nakano E, Watanabe T et al (2020) Epidemiology and practice patterns for male breast cancer in Japan. Cancer Med 9(16):6069–6075 10.1002/cam4.3267 PMC7433825 32613775

[41] Yu E, Stitt L, Vujovic O et al (2015) Male breast cancer prognostic factors versus female counterparts. Cureus 7(10):e355 10.7759/cureus.355 PMC4652922 26623210

[42] Midding E, Halbach SM, Kowalski C et al (2018) Stigmatization in male breast cancer patients. Am J Mens Health 12(6):2194–2207 10.1177/1557988318799025 PMC6199445 30222029

[43] Co M, Lee A, Kwong A (2020) Delayed presentation and psychosocial aspects of male breast cancer. Cancer Med 9(10):3305–3309 10.1002/cam4.2953 PMC7221437 32167660

[44] Halbach SM, Midding E, Ernstmann N et al (2020) Male breast cancer patients’ perspectives on healthcare. Breast Care (Basel) 15(1):22–29 10.1159/000501956 PMC7098323 32231494

[45] Eggemann H, Ignatov A, Smith BJ et al (2013) Tamoxifen versus aromatase inhibitors in male breast cancer. Breast Cancer Res Treat 137(2):465–470 10.1007/s10549-012-2355-3 23224235

[46] Eggemann H, Altmann U, Costa SD, Ignatov A (2018) Survival benefit of endocrine therapy in male breast cancer. J Cancer Res Clin Oncol 144(2):337–341 10.1007/s00432-017-2539-7 PMC11813531 29098396

[47] Fogh S, Hirsch AE, Goldberg SI et al (2011) Tamoxifen with irradiation in hormone receptor–positive male breast cancer. Clin Breast Cancer 11(1):39–45 10.3816/CBC.2011.n.007 21421521

[48] Lin AP, Huang TW, Tam KW (2021) Treatment of male breast cancer: meta-analysis of real-world evidence. Br J Surg 108:znab279 10.1093/bjs/znab279 34476472

[49] Harlan LC, Zujewski JA, Goodman MT, Stevens JL (2010) Male breast cancer in the United States. Cancer 116(15):3558–3568 10.1002/cncr.25153 PMC2910812 20564105

[50] Makdissi FBA, Santos SS, Bitencourt A, Campos FAB (2022) Male breast cancer for urologists. Int Braz J Urol 48(5):760–770 10.1590/S1677-5538.IBJU.2021.0828 PMC9388172 35373955

[51] Yadav S, Karam D, Riaz IB et al (2020) Male breast cancer in the United States: treatment patterns. Cancer 126(1):26–36 10.1002/cncr.32472 PMC7668385 31588557

[52] Leone JP, Leone J, Zwenger AO et al (2015) Prognostic significance of tumor subtypes in male breast cancer. Breast Cancer Res Treat 152(3):601–609 10.1007/s10549-015-3488-y 26126972

[53] Srour MK, Amersi F, Mirocha J et al (2020) Male breast cancer: single-institution experience. Am Surg 86(10):1345–1350 10.1177/0003134820964444 33103460

[54] Liu N, Johnson KJ, Ma CX (2018) Male breast cancer: updated SEER analysis. Clin Breast Cancer 18(5):e997–1002 10.1016/j.clbc.2018.06.013 30007834

[55] Venkitaraman AR (2002) Cancer susceptibility and BRCA function. Cell 108(2):171–182 10.1016/s0092-8674(02)00615-3 11832208

[56] Rizzolo P, Silvestri V, Tommasi S et al (2013) Male breast cancer: genetics and epigenetics. Ann Oncol 24(Suppl 8):viii75–82 10.1093/annonc/mdt316 24131976

[57] Pritzlaff M, Summerour P, McFarland R et al (2017) Male breast cancer in a multigene panel cohort. Breast Cancer Res Treat 161:575–586 10.1007/s10549-016-4085-4 PMC5241330 28008555

[58] Wolpert N, Warner E, Seminsky MF et al (2000) BRCA mutations in male breast cancer in Canada. Clin Breast Cancer 1(1):57–63 10.3816/CBC.2000.n.005 11899391

[59] Basham VM et al (2002) BRCA mutations in male breast cancer. Breast Cancer Res 4(1):1–5 10.1186/bcr419 PMC83848 11879560

[60] Abdelwahab Yousef AJ (2017) Male breast cancer: epidemiology and risk factors. Semin Oncol 44(4):267–272 10.1053/j.seminoncol.2017.11.002 29526255

[61] Rizzolo P, Zelli V, Silvestri V et al (2019) Genetic susceptibility in male breast cancer. Int J Cancer 145:390–400 10.1002/ijc.32106 30613976

[62] Meijers-Heijboer H, van den Ouweland A, Klijn J et al (2002) CHEK2 and breast cancer susceptibility. Nat Genet 31(1):55–59 10.1038/ng879 11967536

[63] Hallamies S, Pelttari LM, Poikonen-Saksela P et al (2017) CHEK2 mutation and male breast cancer risk. BMC Cancer 17(1):1–5 10.1186/s12885-017-3631-8 PMC5584025 28874143

[64] Curia MC, Catalano T, Aceto GM (2020) MUTYH and cancer risk. World J Clin Oncol 11(7):428–449 10.5306/wjco.v11.i7.428 PMC7407923 32821650

[65] Theodoratou E, Campbell H, Tenesa A et al (2010) MUTYH variants and colorectal cancer risk. Br J Cancer 103(12):1875–1884 10.1038/sj.bjc.6605966 PMC3008602 21063410

[66] Rizzolo P, Silvestri V, Bucalo A et al (2018) MUTYH variants in male breast cancer. Front Oncol 8:583 10.3389/fonc.2018.00583 PMC6288482 30564557

[67] Somyajit K, Subramanya S, Nagaraju G (2010) RAD51C and cancer susceptibility. Carcinogenesis 31(12):2031–2038 10.1093/carcin/bgq210 PMC2994284 20952512

[68] Dawson LM, Smith KN, Werdyani S et al (2020) RAD51C pathogenic variant and founder effect. Mol Genet Genomic Med 8(2):e1070 10.1002/mgg3.1070 PMC7005661 31782267

[69] Hu C, Hart SN, Gnanaolivu R et al (2021) Population-based study of breast cancer genes. N Engl J Med 384(5):440–451 10.1056/NEJMoa2005936 PMC8127622 33471974

[70] Spurdle AB, Healey S, Devereau A, Hogervorst FB et al (2012) ENIGMA–evidence-based network for the interpretation of germline mutant alleles: an international initiative to evaluate risk and clinical significance associated with sequence variation in BRCA1 and BRCA2 genes. Hum Mutat 33(1):2–7. 10.1002/humu.21628 Epub 2011 Nov 3 PMC3240687 21990146

