# Supplementary material for: Male breast cancer: clinicopathological features and prevalence of germline pathogenic variants
Source: Breast Cancer Res Treat. 2026 Nov 4;220(1):26. doi: 10.1007/s10549-026-08062-9 (PMC13736371; doi:10.1007/s10549-026-08062-9)
Supplement: Supplementary file 1 [file 10549_2026_8062_MOESM1_ESM.docx]

**Supplementary materials**

**Table S1** - Treatment patterns for patients with localized disease included in the study.

| **Variable** | **n=106 (100%)** |
| --- | --- |
| **Surgery**  Yes*  No | 96 (90,6)  10 (9,4) |
| **Type of Surgery**  Total mastectomy  Segmental resection  NA | 91 (85,8)  4 (3,8)  1 (0,9) |
| **Management of axillary lymph nodes**  Axillary dissection  Sentinel lymph node  NA | 67 (63,2)  27 (25,8)  1 (0,9) |
| **Chemotherapy**  Adjuvant  Neoadjuvant  No | 57 (53,8)  13 (12,3)  25 (23,6) |
| **Chemotherapy protocol**  AC  AC-T  ACdd-T  TC  CMF  Other  NA | 20 (18,9)  25 (23,6)  2 (1,9)  7 (6,6)  5 (4,7)  8 (7,5)  3 (2,8) |
| **Adjuvant radiotherapy**  Yes  No  NA | 51 (48)  40 (37,7)  4 (3,8) |
| **Adjuvant endocrine therapy**  Tamoxifen  Aromatase inhibitor + GnRH analog  NA | 74 (69,8)  3 (2,8)  12 (11,3) |

*One patient with metastatic disease underwent hygienic surgery.

AC: anthracycline/cyclophosphamide; AC-T: anthracycline/cyclophosphamide/taxane; ACdd-T: anthracycline/cyclophosphamide in dense dose/taxanes; TC: docetaxel/cyclophosphamide; CMF: cyclophosphamide/methotrexate/fluorouracil; GnRH: gonadotropin-releasing hormone. NA = No available

**Table S2 -** Comparison of the baseline characteristics of tested vs untested patients

| **Characteristic Tested** | **Tested N = 55¹** | **Not tested N = 51¹** | **p-value²** |
| --- | --- | --- | --- |
| **Treatment Center**  A.C. Camargo  UNICAMP | 44 (80%)  11 (20%) | 27 (53%)  24 (47%) | 0.002 |
|  |  |  |  |
|  |  |  |  |
| **Age** | 60.6 (51.0, 66.6) | 57.2 (49.6, 65.4) | 0.6³ |
| **Family history**  Unknown | 39 (72%)  0 | 29 (58%)  2 | 0.2 |
|  |  |  |  |
| **Histological grade**  Grade 1  Grade 2  Grade 3  Unknown | 1 (2%)  25 (45%)  21 (38%)  8 | 1 (2.0%)  28 (56%)  20 (39%)  2 | >0.9 |
|  |  |  |  |
|  |  |  |  |
|  |  |  |  |
|  |  |  |  |
| **Clinical stage**  I  II  III  IV  Unknown | 19 (35%)  16 (29%)  12 (22%)  5 (9%)  3 | 5 (10%)  19 (37%)  22 (43%)  5 (10%)  0 | 0.008 |
|  |  |  |  |
|  |  |  |  |
|  |  |  |  |
|  |  |  |  |
|  |  |  |  |
| **Molecular subtype**  Luminal  Luminal HER2+  HER2+  Triple-negative  Unknown | 41 (75%)  9 (16%)  1 (2%)  2 (4%)  2 | 42 (82%)  6 (12%)  0 (0%)  3 (6%)  0 | 0.7 |
|  |  |  |  |
|  |  |  |  |
|  |  |  |  |
|  |  |  |  |
|  |  |  |  |
| **chemo_group**  Unknown | 36 (71%)  4 | 34 (68%)  1 | 0.8 |
|  |  |  |  |
| **vital status**  Alive  Dead | 53 (96%)  2 (4%) | 33 (64%)  18 (36%) | <0.001 |
|  |  |  |  |
|  |  |  |  |

Notes:
 ¹ n (%); Median (Q1, Q3)
 ² Fisher`s test;

³ Wilcoxon test.

**Table S3** - Univariate/multivariate analysis of factors associated with overall mortality.

| **Variable** | **Mean survival, months** | **Five years overall survival, %** | ***p*** | **RR (IC 95%)** | **Adjusted p** |
| --- | --- | --- | --- | --- | --- |
| **Age**  < 60 years  ≥ 60 years | 182  217 | 76,3  87,1 | 0,62 |  |  |
| **Histologic grade**  Grade 2  Grade 3 | -  - | 92,2  64,6 | 0,005 | 7,6 (1,6 – 34,7) | 0,009 |
| **Stage T**  T1  T2  T3  T4 | 241  190  49  97 | 97,2  82,3  50  55,8 | 0,0001 |  |  |
| **Stage N**  N0  N+ | 252  131 | 93,2  70,1 | 0,02 | 6,4 (1,4 – 29,3) | 0,017 |
| **Stage M**  M0  M1 | 222  40 | 84  27,8 | 0,002 |  |  |
| **Molecular subtype**  Luminal  Luminal HER2/HER2 positive  Triple negative | 187  153  122 | 82,9  93,8  40 | 0,091 |  |  |
| **Ki67**  < 20%  ≥ 20% | 108  121 | 88,3  87,6 | 0,784 |  |  |
| **Estrogen receptor**  Negative  Positive | 156  189 | 53,3  83,2 | 0,223 |  |  |
| **Progesterone receptor**  Negative  Positive | 139  175 | 49,7  85,7 | 0,079 |  |  |
| **HER2 expression**  Positive  Negative | 154  212 | 94,4  82,9 | 0,435 |  |  |
| **Neoadjuvant/adjuvant chemotherapy**  Yes  No | 190  163 | 88,1  70,8 | 0,007 | 8,7 (2,7 – 28,8) | 0,0001 |

**Table S4** - Surgical approach to male breast cancer in different studies.

| **Author, year, number of patients** | **Mastectomy (%)** | **Conservative surgery (%)** |
| --- | --- | --- |
| **Srour et al., 2020, 49**^66^ | 87,8 | 4,1 |
| **Yadav et al., 2018, 81**^64^ | 86,0 | 14,0 |
| **Leone et al, 2016, 1263**^65^ | 81,2 | 17,6 |
| **This study, 2023, 106** | 85,8 | 3,8 |

Source: Adapted from Makdissi et al., 2023^63^

**Table S5** - Studies that performed multigene panel tests in men with breast cancer.

| **Author, year** | **Cohort** | **Panel size** | **Detection rate** | **Gene with pathogenic variant (%)** |
| --- | --- | --- | --- | --- |
| Tedaldi et al, 2020^47^ | 70 patients selected during genetic counseling | 94 genes | 21,4% | *BRCA2* (8,6), *BRCA1* (4,3), *PALB2* (1,4), *CHEK2* (1,4), *ATM* (1,4), *RAD51C* (1,4), *BAP1* (1,4), *EGFR* (1,4) |
| Gaddam et al, 2020^48^ | 414Men who completed the panel for various clinical indications. Eighteen patients had a personal history of breast cancer. | Various commercial panels | 27,8% | *BRCA2* (16,7), *NBN* (5,6), *BARD1* (5,6) ^a^ |
| Scarpitta et al, 2019^49^ | 81 patients selected during genetic counseling | 24 genes | 18,5% | *BRCA2* (13,6), *BRIP1* (2,5), *MUTYH* (1,2),  *PMS2* (1,2) |
| Rizzolo et al, 2019^67^ | 523 patients, not selected based on age at diagnosis or family history of cancer | 50 genes | 9,0% | *BRCA2* (2,9), *PALB2* (1,1), *BRCA1* (1,0); *ATM* (0,6); *APC* (0,4), *BARD1* (0,4), *BLM* (0,4), *CHEK2* (0,4), *FANCM* (0,2), *RAD51D* (0,4), *CASP8* (0,2), *EPCAM* (0,2), *MUTYH* (0,2), *NF1* (0,2), *RAD50* (0,2), *RAD51C* (0,2) |
| Fostira et al, 2018^50^ | 102 patients not selected based on age at diagnosis or family history of cancer | 94 genes | 12,7% | *BRCA2* (6,9), *ATM* (2,0), *BRCA1* (1,0), *CHEK2* (1,0), *PMS2* (1,0), *FANCL* (1,0) |
| Vogel Postula et al, 2018^51^ | 381 patients | 8 to 32 genes | 12,1% | *BRCA2* (5,5); *CHEK2* (4,5); *PALB2* (1,0); *BRCA1* (1,0); *ATM* (0,5) ^b^ |
| Pritzlaff et al, 2017^21^ | 708 patients (538 caucasians or Ashkenazi Jews) | 16 genes | 18,1% | *BRCA2* (8,1), *CHEK2* (3,8), *ATM* (1,0), *BRCA1* (0,9), *PALB2* (0,8), *NF1* (0,6), *BARD1* (0,4), *BRIP1* (0,2), *MRE11A* (0,2), *NBN* (0,2), *RAD51D* (0,2) |
| Susswein et al, 2016^52^ | 10.030 individuals (men and women) referred for evaluation by a hereditary cancer panel. Included 51 men with a history of breast cancer. | Various commercial panels (up to 29 genes) | 11,8% | *CHEK2* (7,8), *BRCA2* (2,0), *PALB2* (2,0), *BRCA1* (2,0) |
| Tung et al, 2015^11^ | 2.158 Individuals (men and women). Included 22 men with a personal history of breast cancer. | 25 genes | 31,8% | *BRCA2* (13,6); *CHEK2* (4,5); *PALB2* (4,5); *BRCA1* (4,5); *ATM* (4,5) ^c^ |
| This study, 2023 | 106 individuals, with 55 evaluated using a germline panel. | Panels ranging from 144 to 26 genes | 21,8% | *BRCA2* (11), *CHEK2* (5,5), *MUTYH* (3,6), *RAD51C* (1,8) |

a – The data refers only to the 18 patients with a history of breast cancer; b – other genes not reported; c – The data refers only to the 22 men with a history of breast cancer.

**Table S6** - Selected studies evaluating clinicopathological characteristics in men with breast cancer and altered and wild-type BRCA1/2.

|  | **Pathogenic variant in BRCA1** | | | **Pathogenic variant in BRCA2** | | | ***wild type BRCA*** | | |
| --- | --- | --- | --- | --- | --- | --- | --- | --- | --- |
| Author, year  (total number of participants) | Ottini, 2012 (378) | Deb, 2012  (60) | Gargiulo, 2016 (47) | Ottini, 2012  (378) | Deb, 2012  (60) | Gargiulo, 2016 (47) | Ottini, 2012  (378) | Deb, 2012  (60) | Gargiulo, 2016 (47) |
| Number of patients/Total evaluated (%) | 4/378 (1,1) | 3/60 (5) | 1/17 (5,9) | 46/378 (12,2) | 25/60 (41,6) | 5 (29,4) | 328/378 (86,7) | 32/60 (53,3) | 10/17 (58,8) |
| Average/median age (years) | 62 / NI | NI / 65,6 | NI / 40 | 58,9 / NI | NI / 61 | NI / 72 | NI / NI | NI / 63,2 | NI / 61 |
| FH of CA breast/ovary (%) | 3 (75) | 3 (100) | 1 (100) | 31 (67,4) | 25 (100) | 2 (40) | 105 (32) | 32 (100) | 5 (50) |
| Personal history of other malignant neoplasms (%) | 0 | 0 (0) | 1 (100) | 12 (26,1)^1^ | 5 (8,3)^2^ | 2 (40) | 44 (13,4) | 5 (8,3) | 2 (20) |
| BC Contralateral(%) | 0 | 0 (0) | NI | 7 (15,2) | 1 (1,6) | NI | 9 (2.7) | 1 (1,6) | NI |
| Histology (%)  Invasive ductal carcinoma  Ductal carcinoma in situ  Invasive lobular carcinoma  Others | (n = 4)  4 (100)  0 (0)  0 (0)  0 (0) | (n = 3)  2 (66,6)  0 (0)  0 (0)  1 (33,3) | (n = 1)  1 (100)  0 (0)  0 (0)  0 (0) | (n = 34)  30 (88,3)  2 (5,9)  0 (0)  2 (5,8) | (n = 25)  24 (96)  0 (0)  0 (0)  1 (4) | (n = 5)  5 (100)  0 (0)  0 (0)  0 (0) | (n = 254)  220 (86,6)  20 (7,9)  4 (1,6)  10 (3,9) | (n = 34)  30 (88,2)  0 (0)  2 (6,3)  2 (6,3) | (n = 10)  10 (100)  0 (0)  0 (0)  0 (0) |
| Immunohistochemistry (%)  Rh positive  HER2 positive  Triple negative | (n = 4)  3 (75)  0 (0)  1 (25) | (n = 3)  3 (100)  0 (0)  0 (0) | (n = 1)  1 (100)  0 (0)  0 (0) | (n = 19)  16 (84,2)  3 (15,8)  0 (0) | (n = 25)  23 (92)  2 (8)  0 (0) | (n = 5)  5 (100)  1 (20)  0 (0) | (n = 166)  159 (95,8)  1 (0,6)  6 (3,6) | (n = 30)  26 (86,7)  3 (10)  1 (3,3) | (n = 9)  9 (100)  1 (11)  0 (0) |
| histological grade (%)  1-2  3 | (n = 3)  1 (33,3)  2 (66,7) | (n = 3)  0 (0)  3 (100) | NI | (n = 31)  14 (45,2)  17 (54,8) | (n = 25)  13 (52)  12 (48) | NI | (n = 227)  169 (74,4)  58 (25,6) | (n = 32)  20 (62,5)  12 (37,5) | NI |
| Staging(%)  I – II  III – IV | (n = 3)  2 (66,7)  1 (33,3) | NI | (n = 1)  1 (100)  0 (0) | (n = 24)  15 (62,5)  9 (37,5) | NI | (n = 4)  2 (40)  2 (40) | (n = 187)  151 (80,7)  36 (19,3) | NI | (n = 10)  9 (90)  1 (10) |
| Compromised lymph nodes (%) | (n = 3)  2 (66,7) | (n = 3)  2 (66,7) | (n = 1)  1 (100) | (n = 30)  17 (56,7) | (n = 20)  9 (45) | (n = 5)  2 (40) | (n = 209)  80 (38,3) | (n = 23)  9 (39,1) | (n = 10)  5 (50) |
| NI: Not informed; FH: Family history; CA: cancer; BC: Breast cancer; HR: Hormonal receptor; HER2: human epidermal growth factor receptor 2  ^1, 2^: predominantly prostate cancer (58% and 50%, respectively) | | | | | | | | | |

**APPENDICES**

**Appendix 1 – Detailed description of the genes evaluated in each germline panel used in the study:**

- **26 genes panel**

*APC, ATM, BARD1, BRCA1, BRCA2, BRIP1, CDH1, CHEK2, EPCAM, FAM175A, MLH1, MRE11, MSH2, MSH6, MUTYH, NBN, PALB2, PIK3CA, PMS2, PTEN, RAD50, RAD51C, RAD51D, STK11, TP53, XRCC2.*

- **27 genes panel**

*APC, ATM, BARD1, BMPR1A, BRCA1, BRCA2, BRIP1, CDH1, CDK4, CDKN2A, CHEK2, EPCAM, MLH1, MSH2, MUTYH, MSH6, NBN, PALB2, PMS2, PTEN, RAD51C, RAD51D, RET, SMARCA4, STK11, TP53, NF1.*

- **94 Genes panel**

*AIP, ALK, APC, ATM, BAP1, BLM, BMPR1A, BRCA1, BRCA2, BRIP1, BUB1B, CDC73, CDH1, CDK4, CDKN1C, CDKN2A, CEBPA, CEP57, CHEK2, CYLD, DDB2, DICER1, DIS3L2, EGFR, EPCAM, ERCC2, ERCC3, ERCC4, ERCC5, EXT1, EXT2, EZH2, FANCA, FANCB, FANCC, FANCD2, FANCE, FANCF, FANCG, FANCI, FANCL, FANCM, FH, FLCN, GATA2, GPC3, HNF1A, HRAS, KIT, MAX, MEN1, MET, MLH1, MSH2, MSH6, MUTYH, NBN, NF1, NF2, NSD1, PALB2, PHOX2B, PMS1, PMS2, PRF1, PRKAR1A, PTCH1, PTEN, RAD51C, RAD51D, RB1, RECQL4, RET, RHBDF2, RUNX1, SBDS, SDHAF2, SDHB, SDHC, SDHD, SLX4, SMAD4, SMARCB1, STK11, SUFU, TMEM127, TP53, TSC1, TSC2, VHL, WRN, WT1, XPA, XPC.*

- **112 genes panel**

*ACD, AIP, AKT1, ALK, APC, ATM, AXIN2, BAP1, BARD1, BLM, BMPR1A, BRCA1, BRCA2, BRIP1, BUB1B, CASR, CDC73, CDH1, CDK4, CDKN1B, CDKN1C, CDKN2A, CEBPA, CHEK2, CREBBP, CTNNA1, DICER1, DIS3L2, EGFR, EGLN1, EPCAM, ERBB2, ERCC2, EXT1, EXT2, FANCC, FANCG, FANCM, FH, FLCN, GALNT12, GATA2, GREM1, HNF1A, HOXB13, HRAS, KIF1B, KIT, LZTR1, MAX, MC1R, MEN1, MET, MITF, MLH1, MLH3, MRE11, MSH2, MSH3, MSH6, MUTYH, NBN, NF1, NF2, NSD1, NTHL1, PALB2, PDGFRA, PHOX2B, PMS2, POLD1, POLE, POLH, POT1, PRKAR1A, PRSS1, PTCH1, PTEN, RAD50, RAD51C, RAD51D, RB1, RECQL4, RET, RHBDF2, RUNX1, SDHA, SDHAF2, SDHB, SDHC, SDHD, SLX4, SMAD4, SMARCA4, SMARCB1, SMARCE1, STK11, SUFU, TERC, TERF2IP, TERT, TMEM127, TP53, TSC1, TSC2, VHL, WRN, WT1, XPA, XPC, XRCC2, YAP1.*

- **113 genes panel**

*AKT1, ALK, APC, ATM, AXIN2, BAP1, BARD1, BLM, BMPR1A, BRCA1, BRCA2, BRIP1, BUB1, BUB1B, BUB3, CASR, CDC73, CDH1, CDK4, CDKN1B, CDKN1C, CDKN2A, CHEK2, CREBBP, CTNNA1, CTR9, CYLD, DDB2, DICER1, DIS3L2, EGLN1, ENG, EP300, EPCAM, ERCC2, ERCC3, ERCC4, ERCC5, EXT1, EXT2, EZH2, FANCC, FANCG, FANCM, FH, FLCN, GALNT12, GPC3, GREM1, HNF1A, HOXB13, HRAS, KIF1B, KIT, LZTR1, MAX, MC1R, MEN1, MET, MITF, MLH1, MSH2, MSH3, MSH6, MSR1, MTAP, MUTYH, NBN, NF1, NF2, NSD1, NTHL1, PALB2, PDGFRA, PHOX2B, PMS2, POLD1, POLE, POLH, POT1, PRKAR1A, PTCH1, PTEN, RAD50, RAD51C, RAD51D, RB1, RECQL4, REST, RET, RHBDF2, SDHA, SDHAF2, SDHB, SDHC, SDHD, SLX4, SMAD4, SMARCA4, SMARCB1, SMARCE1, STK11, SUFU, TERT, TMEM127, TP53, TSC1, TSC2, VHL, WRN, WT1, XPA, XPC.*

**Appendix 2 – List of variants of uncertain significance found in the 55 patients evaluated for germline variants.**

| **Panel size in number of genes** | **Pathogenic/probably pathogenic variants** | **Variant of uncertain meaning** |
| --- | --- | --- |
| **27** |  | NM_002485.4(NBN):c.425A>G (p.Asn142Ser) |
| **94** |  | NM_000551.3(VHL):c.463+4C>T |
| **94** | NM_058216.3(RAD51C):c.93del (p.Phe32fs) | NM_000057.4(BLM):c.599C>A; p.(Thr200Lys) \| NM_004364.4(CEBPA):c.1018G>A (p.Gly340Ser) |
| **27** |  | NM_000249.3(MLH1):c.91_92delGCinsTG (p.Ala31Cys) \| NM_000249.3(MLH1):c.1360G>C (p.Gly454Arg) |
| **27** |  | NM_000051.3(ATM):c.95G>A (p.Arg32His) |
| **27** |  | NM_000059.4(BRCA2):c.811G>A (p.Gly271Arg) |
| **27** |  | NM_000051.4(ATM):c.1516G>T (p.Gly506Cys) |
| **26** |  | NM_002878.3(RAD51D):c.26G>C (p.Cys9Ser) |
| **27** |  | NM_000059.3(BRCA2):c.4549A>G (p.Lys1517Glu) |
| **26** |  | NM_004360.5(CDH1):c.2269G>A (p.Glu757Lys) |
| **26** | NM_007194.4(CHEK2):c.1427C>T (p.Thr476Met) - risco | NM_004360.5(CDH1):c.949T>C (p.Phe317Leu) |
| **26** |  | NM_000179.3(MSH6):c.899G>A (p.Arg300Gln) |
| **26** | NM_000059.3(BRCA2):c.2808_2811del (p.Ala938Profs) | NM_000059.4(BRCA2):c.6988A>G (p.Ile2330Val) |
| **26** |  | NM_024675.4(PALB2):c.2834+4T>C |
| **26** |  | NM_000051.4(ATM):c.200A>G (p.Tyr67Cys) |
| **26** | NM_000059.3(BRCA2):c.2812_2815del (p.Ala938fs) | NM_000059.3(BRCA2):c.3220A>T (p.Ser1074Cys); NM_007194.4(CHEK2):c.542G>A (p.Arg181His) |
| **27** |  | NM_000535.7(PMS2):c.2068A>C (p.Lys690Gln) \| NM_000051.4(ATM):c.6067G>A (p.Gly2023Arg) |
| **27** |  | NM_000249.3(MLH1):c.359C>G (p.Ala120Gly) \| NM_002485.4(NBN):c.2029G>A (p.Asp677Asn) |
| **27** |  | NM_002485.5(NBN):c.511A>G (p.Ile171Val) |
| **26** | NM_001048174.2(MUTYH):c.452A>G (p.Tyr151Cys) | NM_000059.4(BRCA2):c.811G>A (p.Gly271Arg) |
| **144** |  | BMPR1A  c.103A>G; p.Met35Val*  FANCD2 c.3849+5_3849+8delinsA*  MITF c.31C>T; p.Pro11Ser^&^ |

*Findings in heterozygosity; & Findings in homozygosity;
